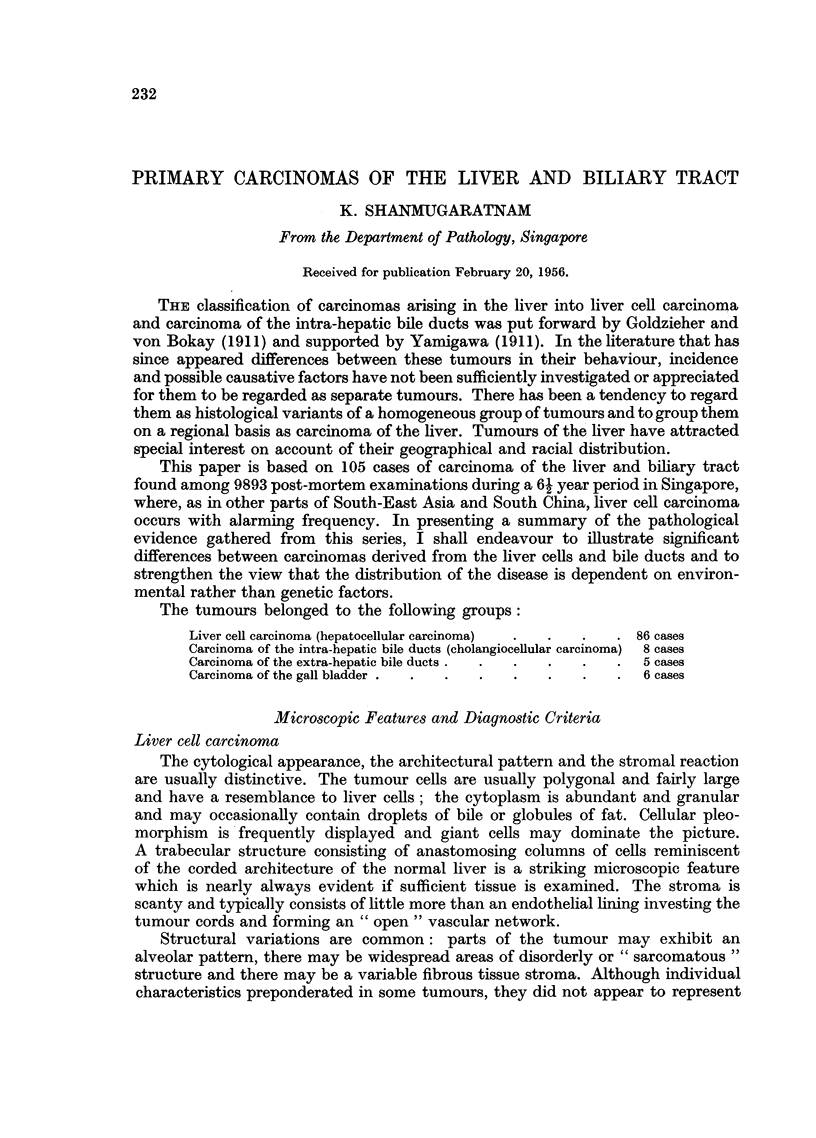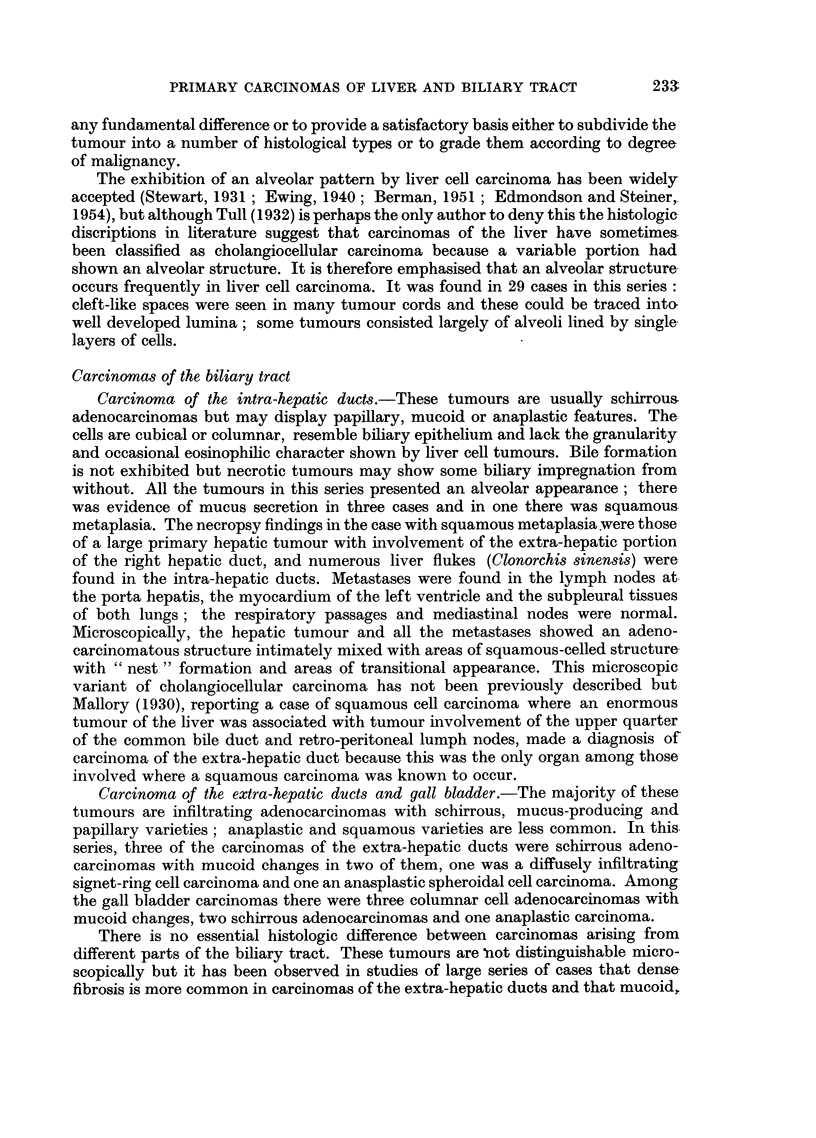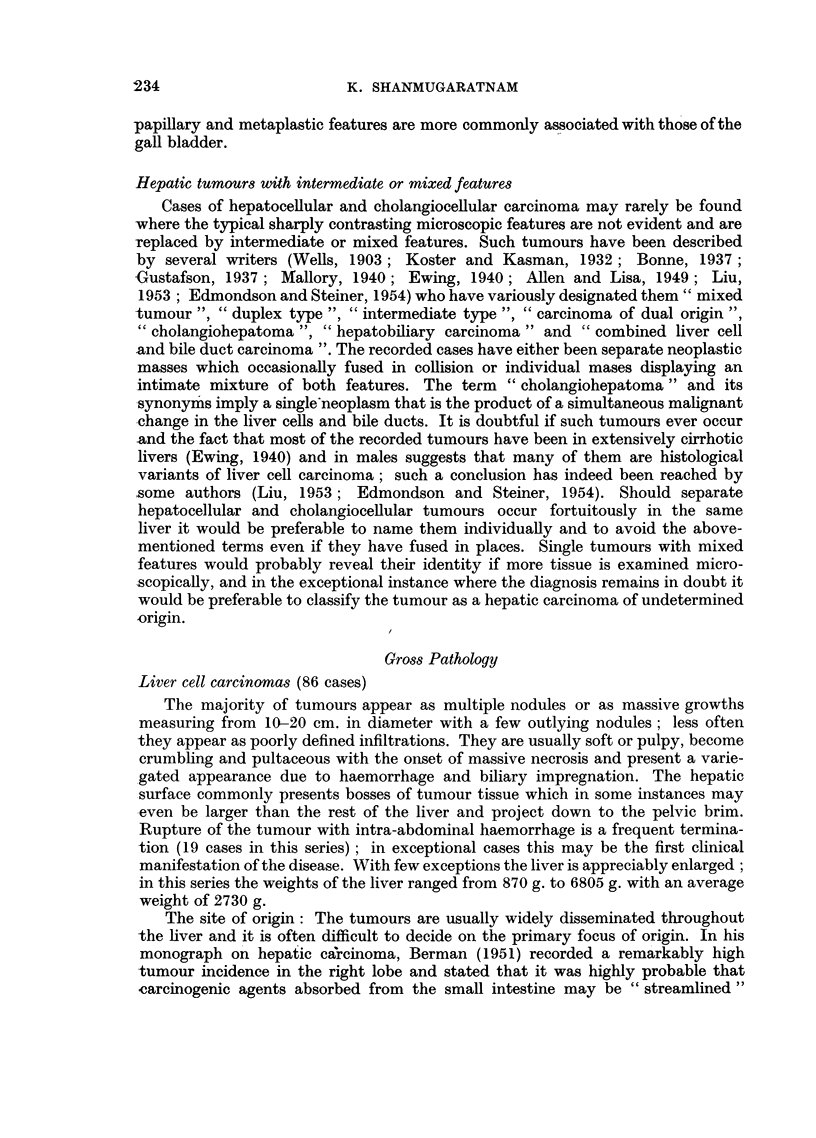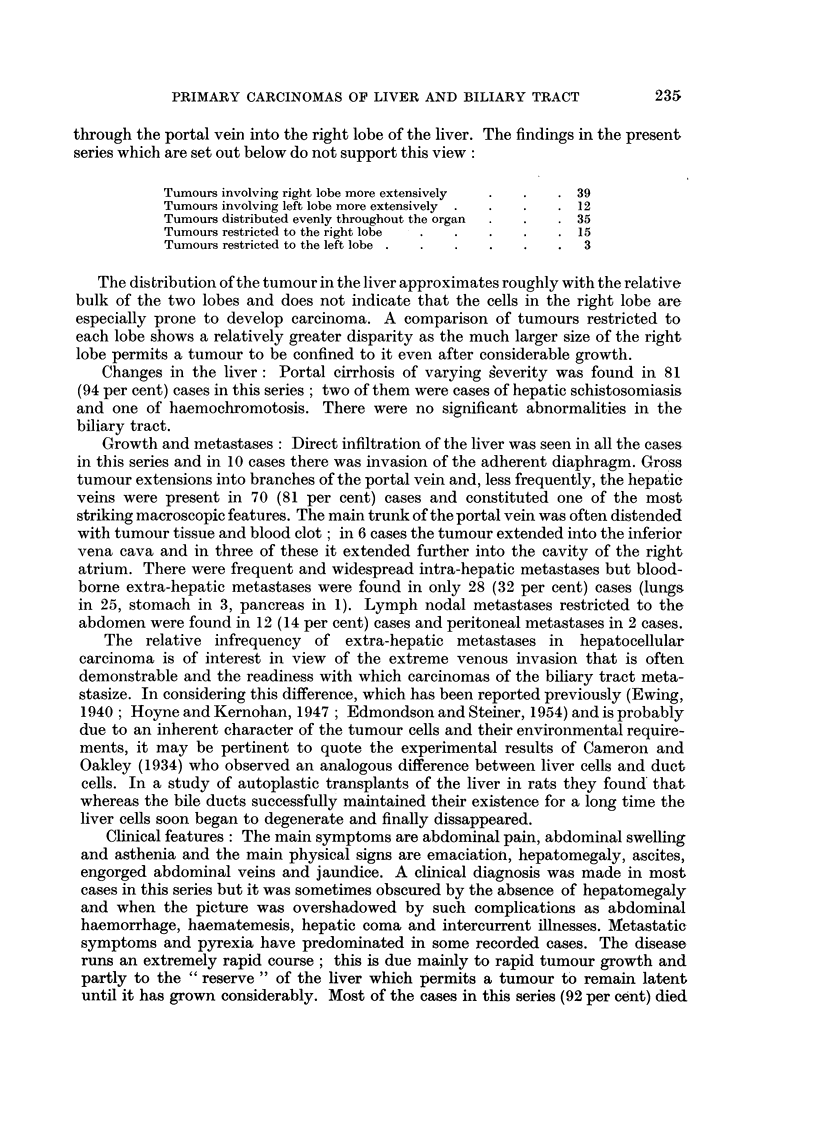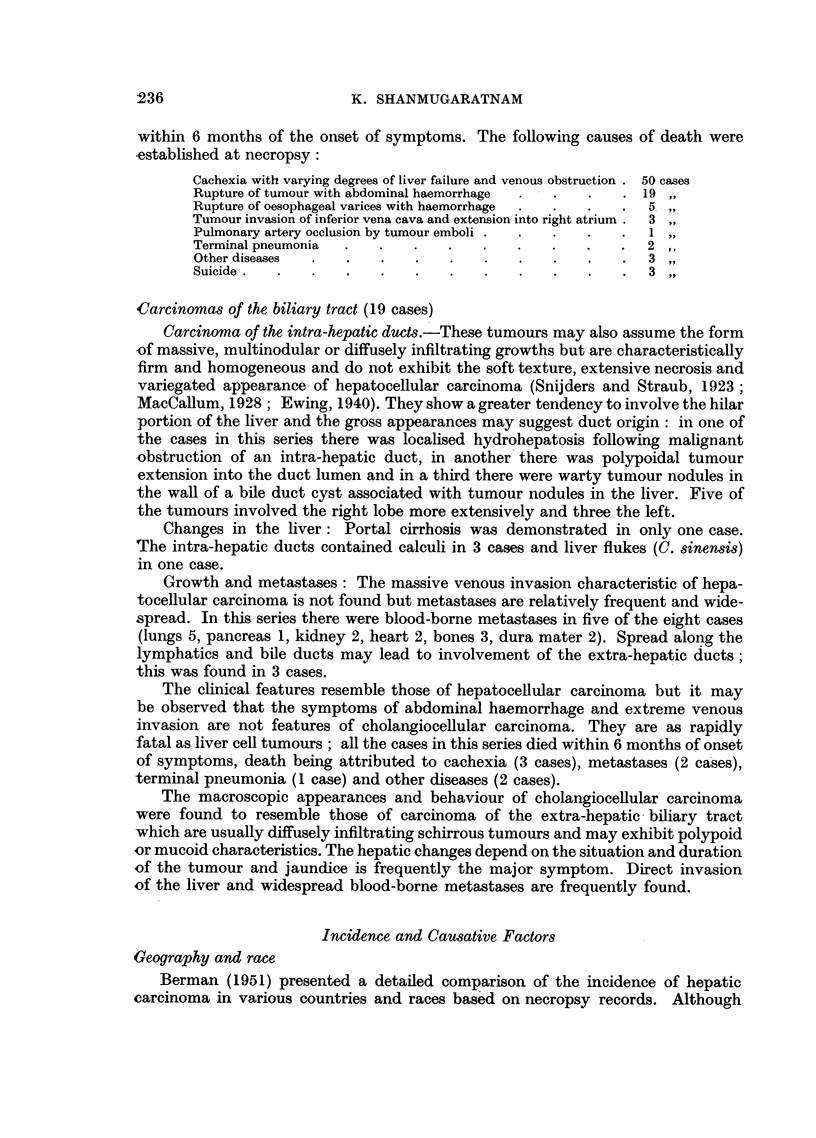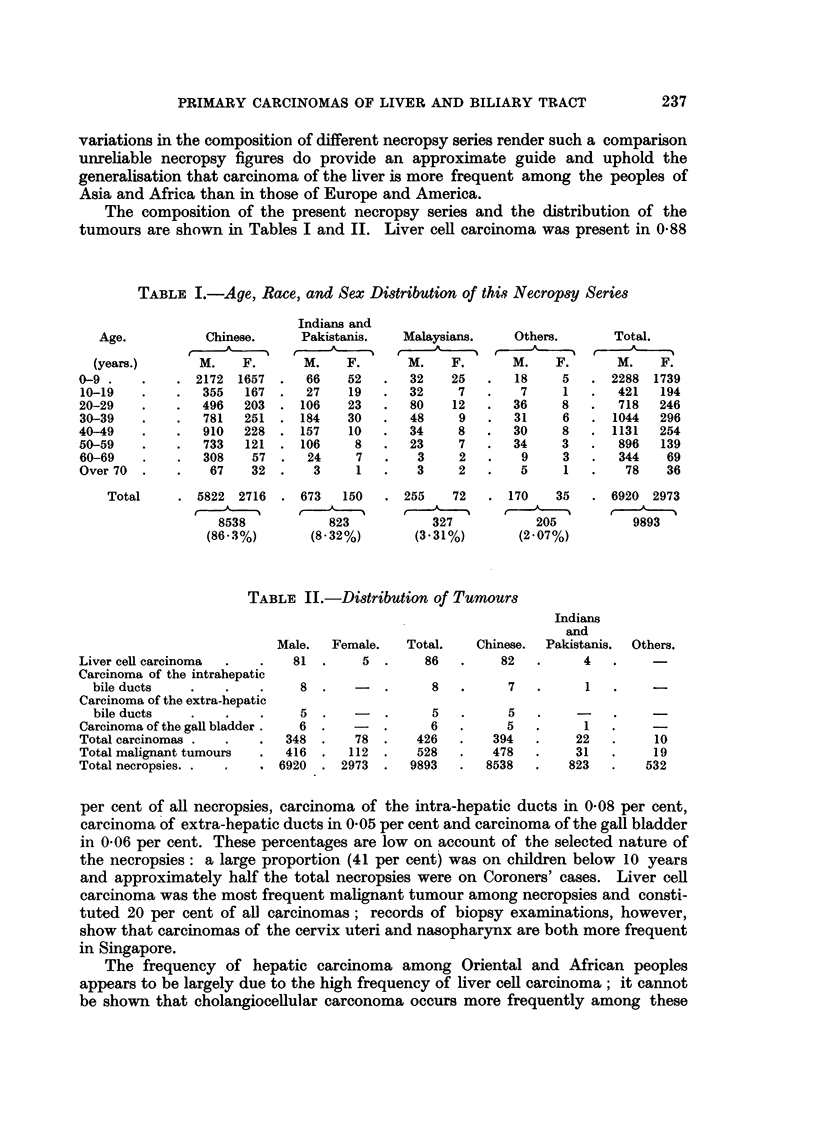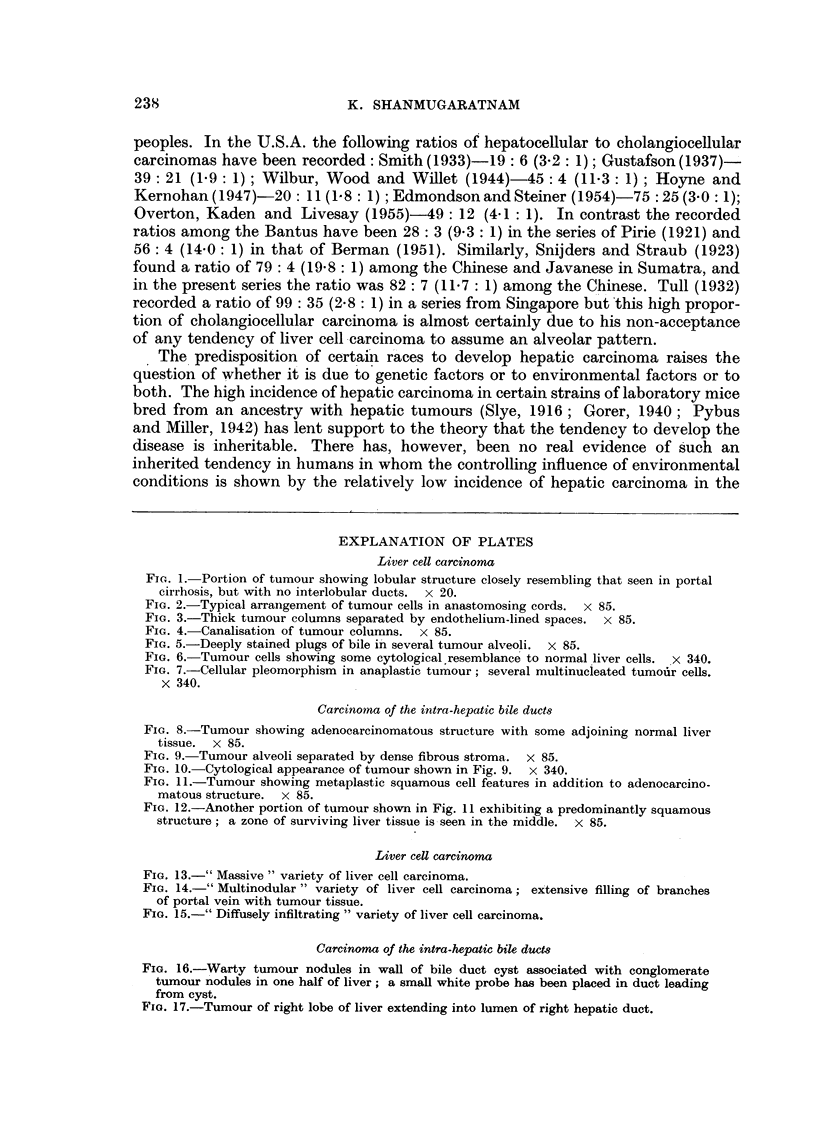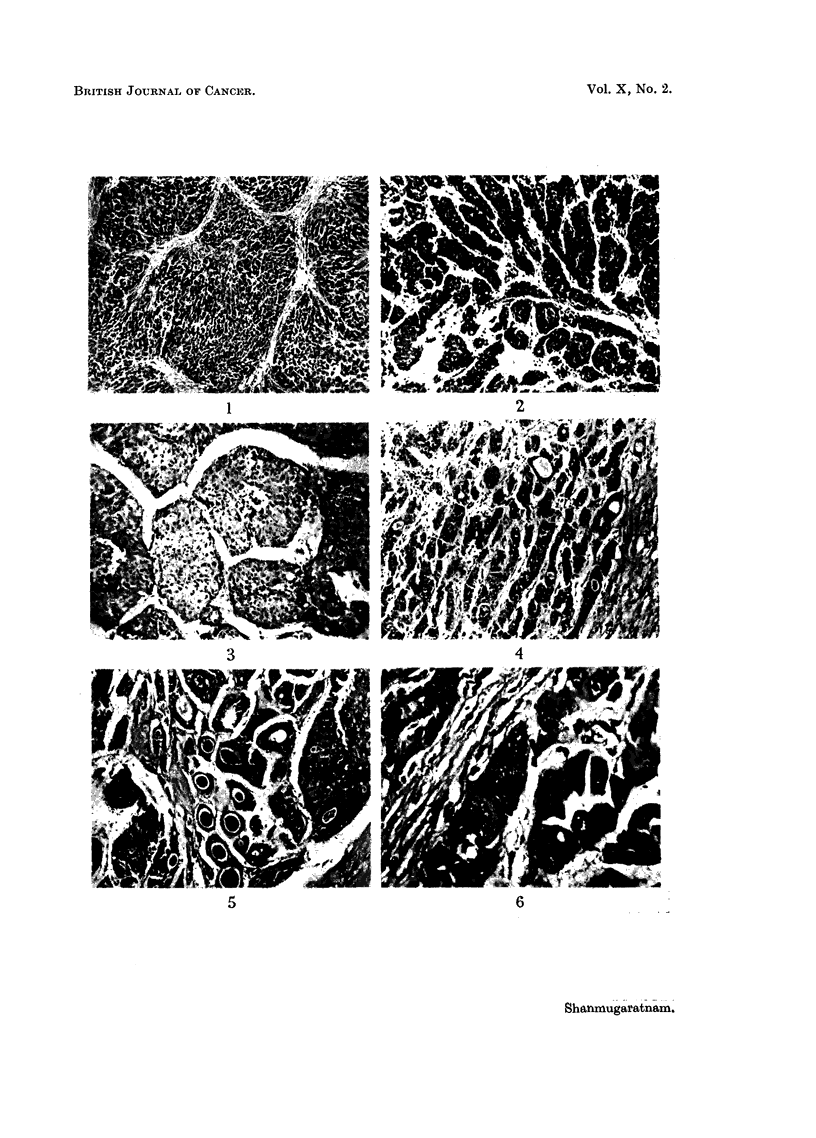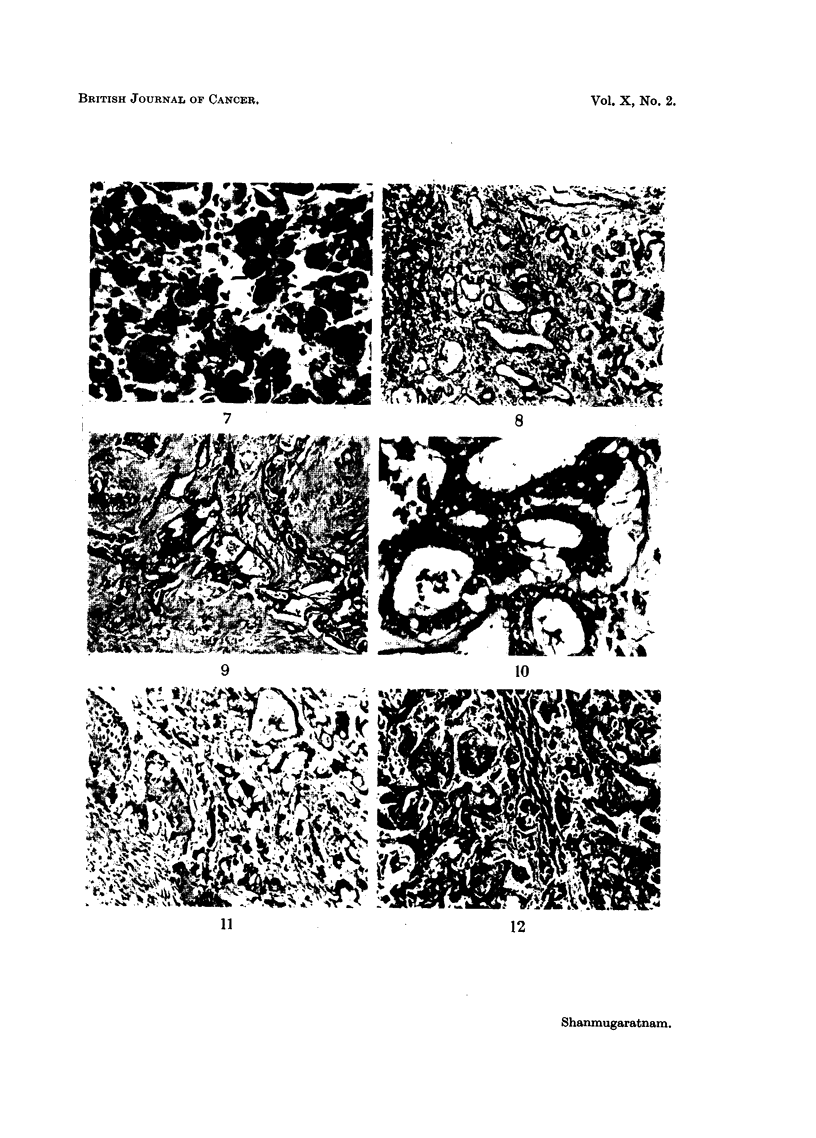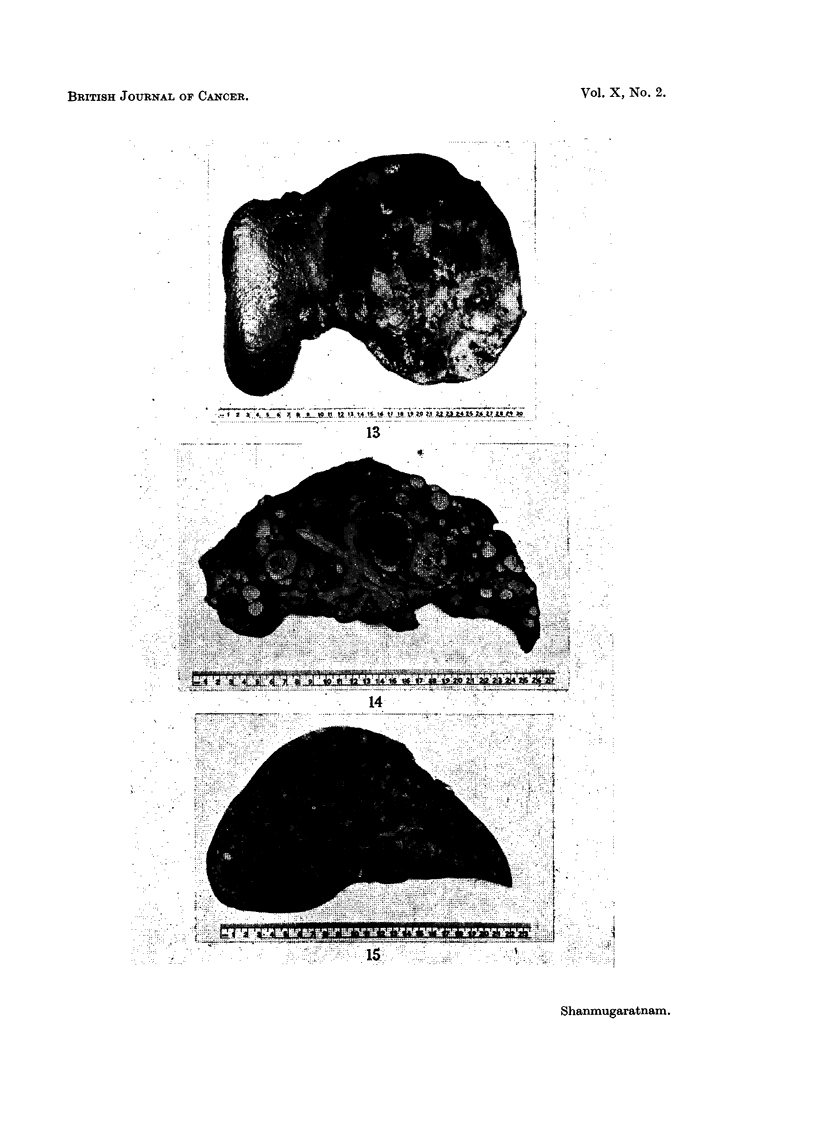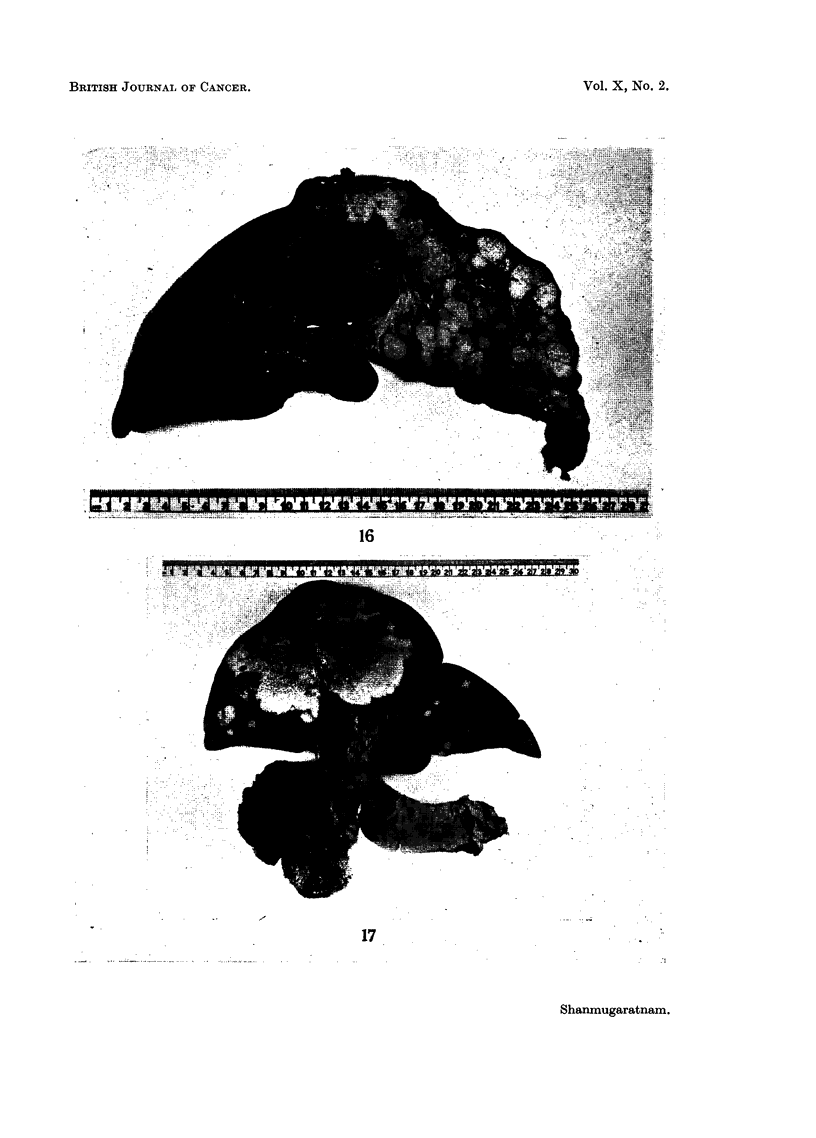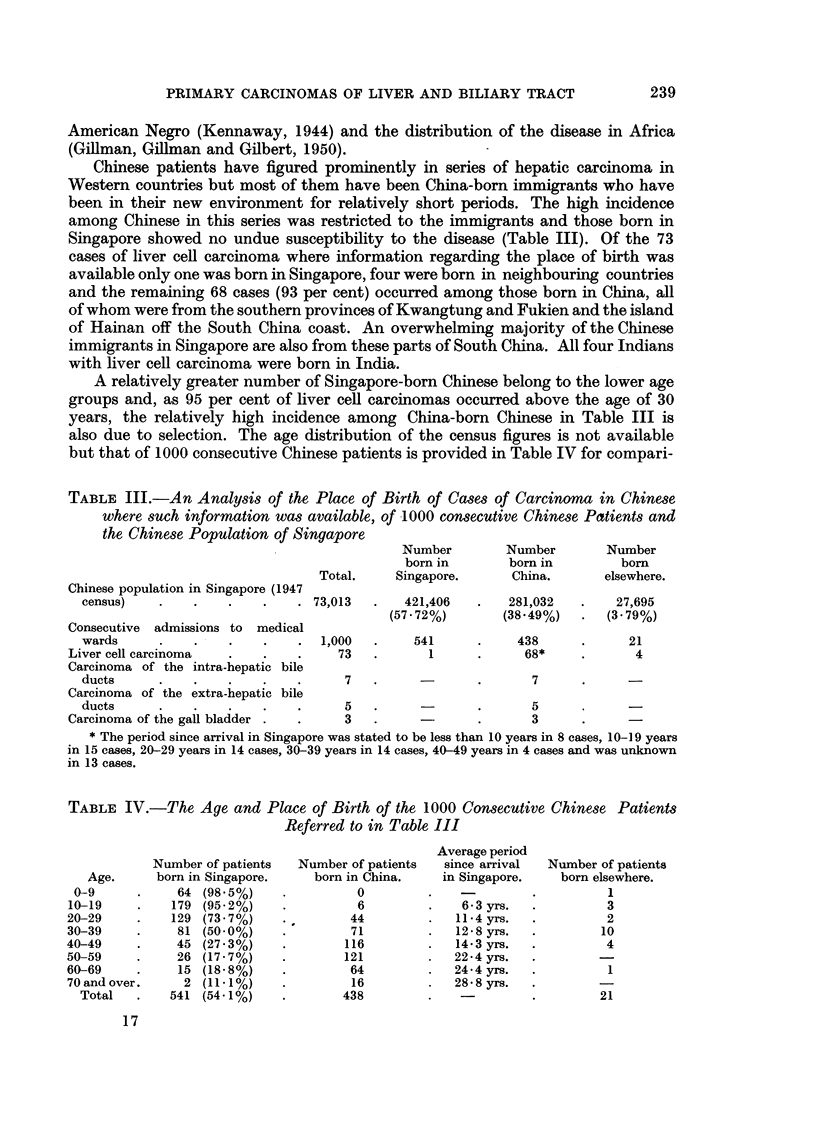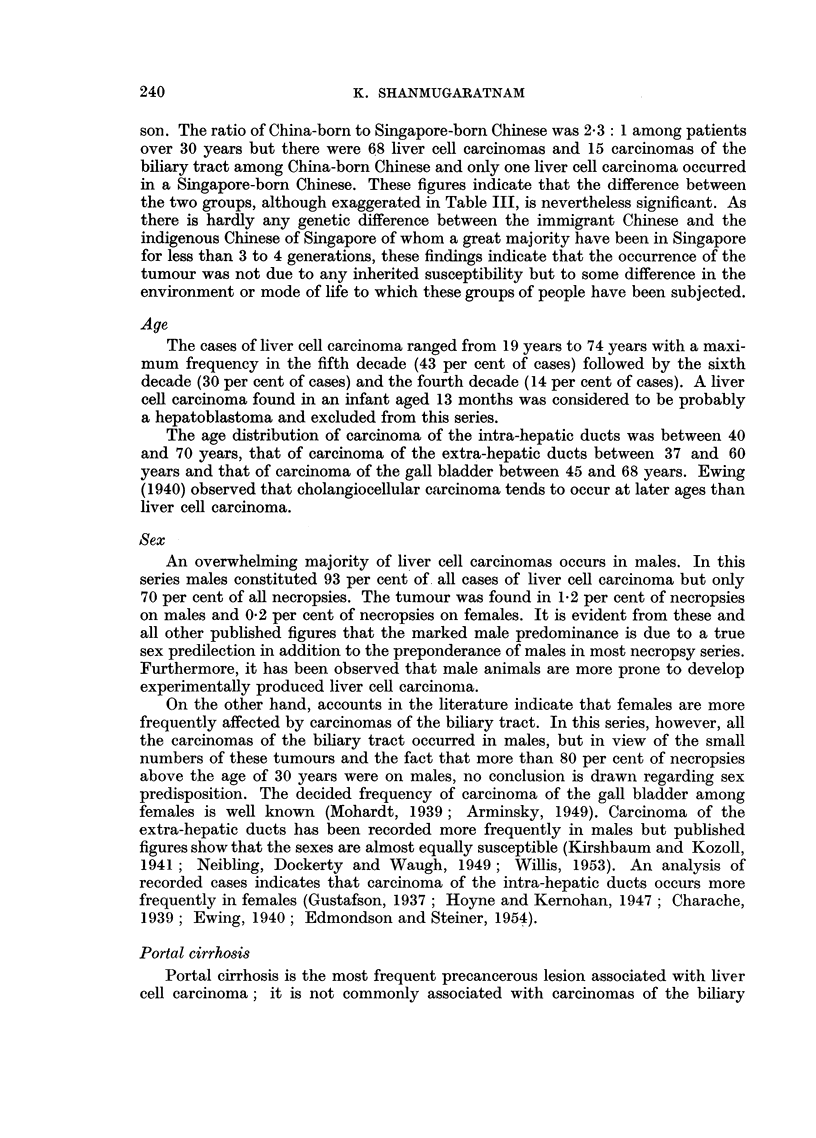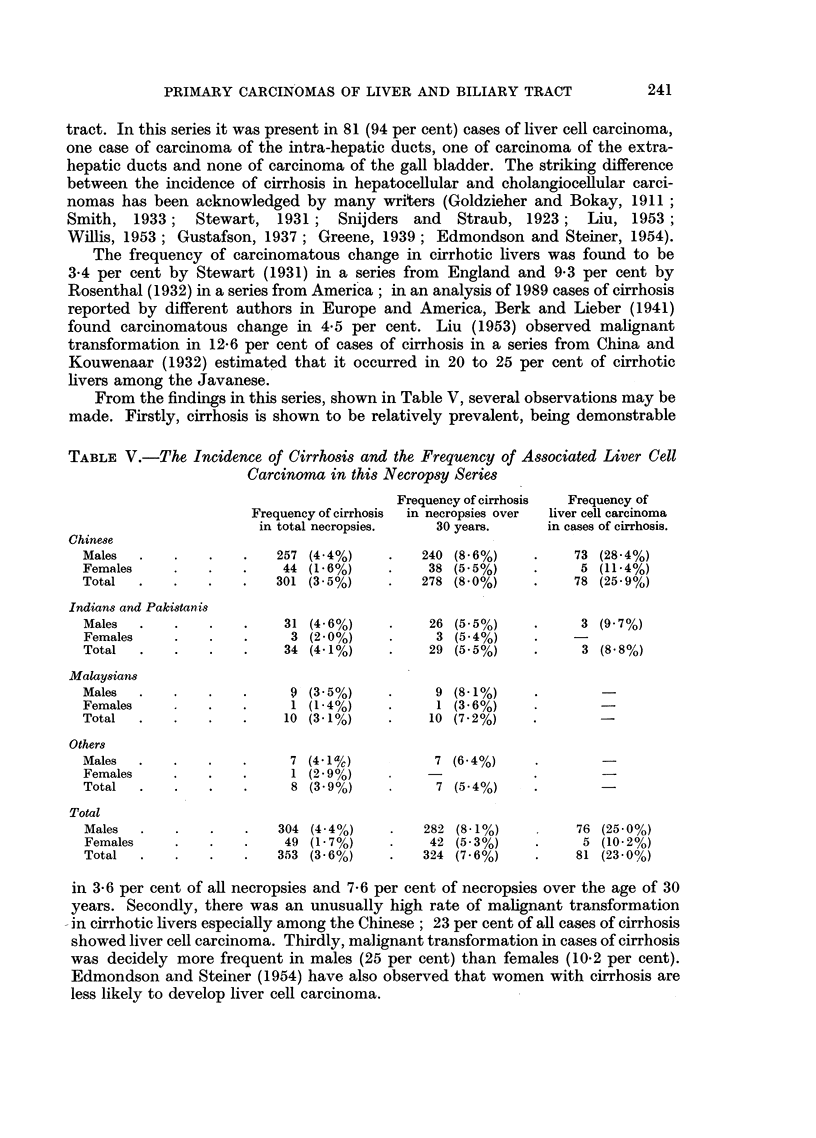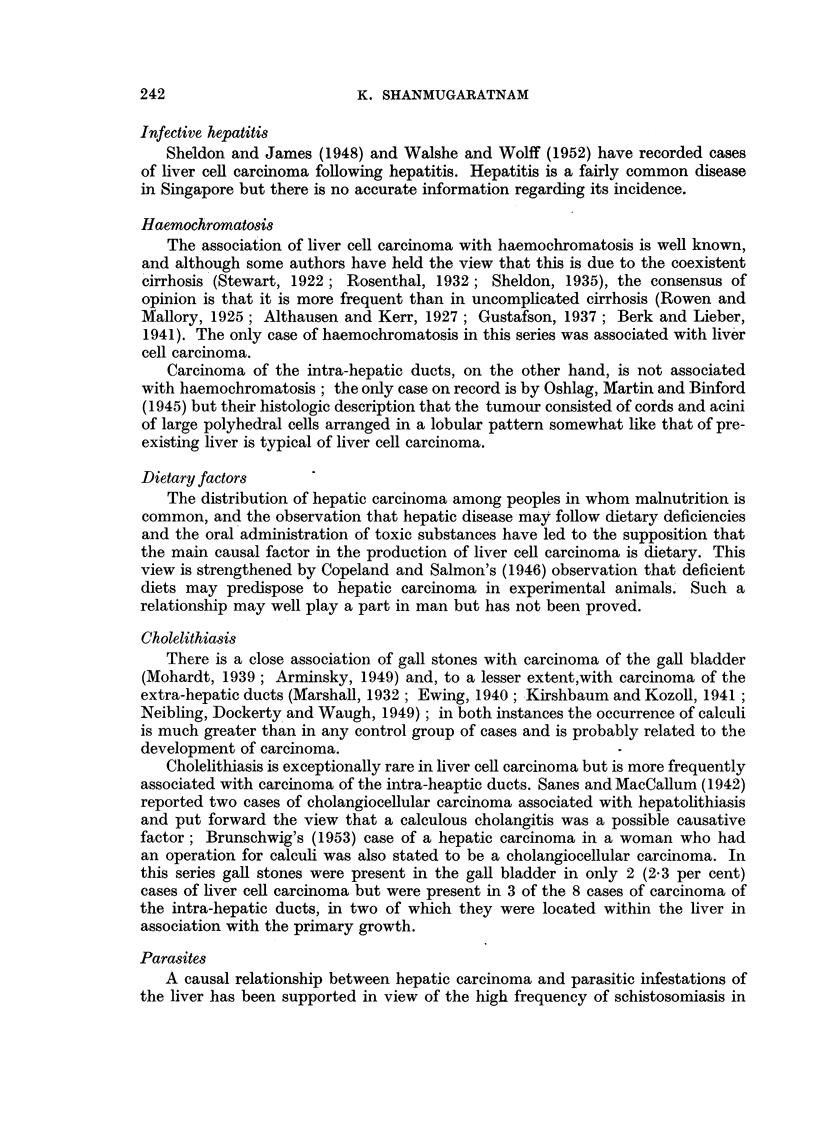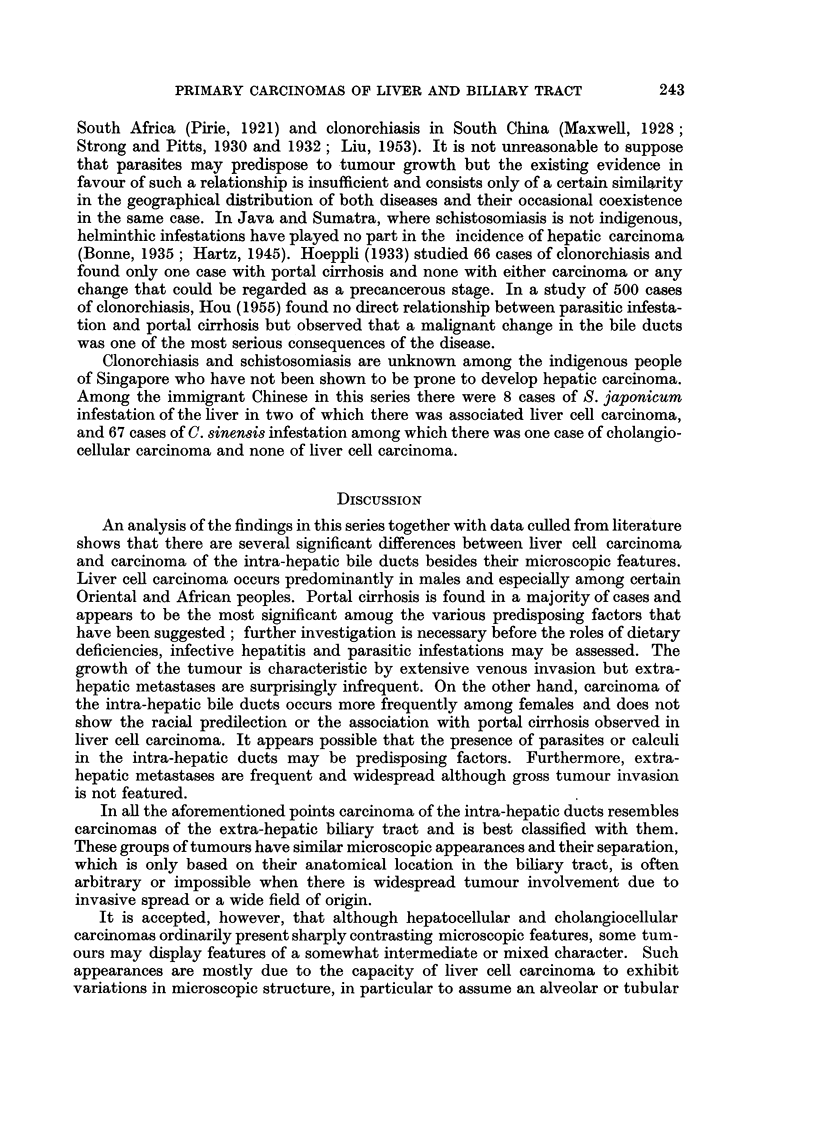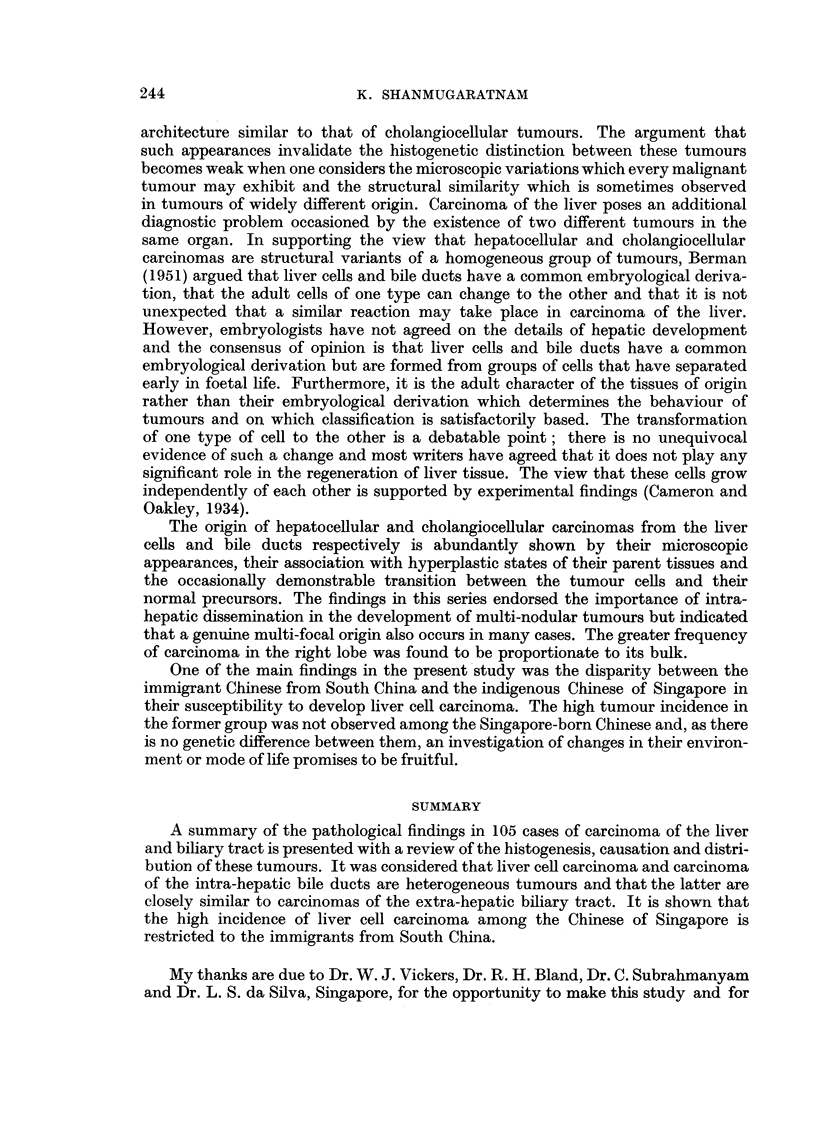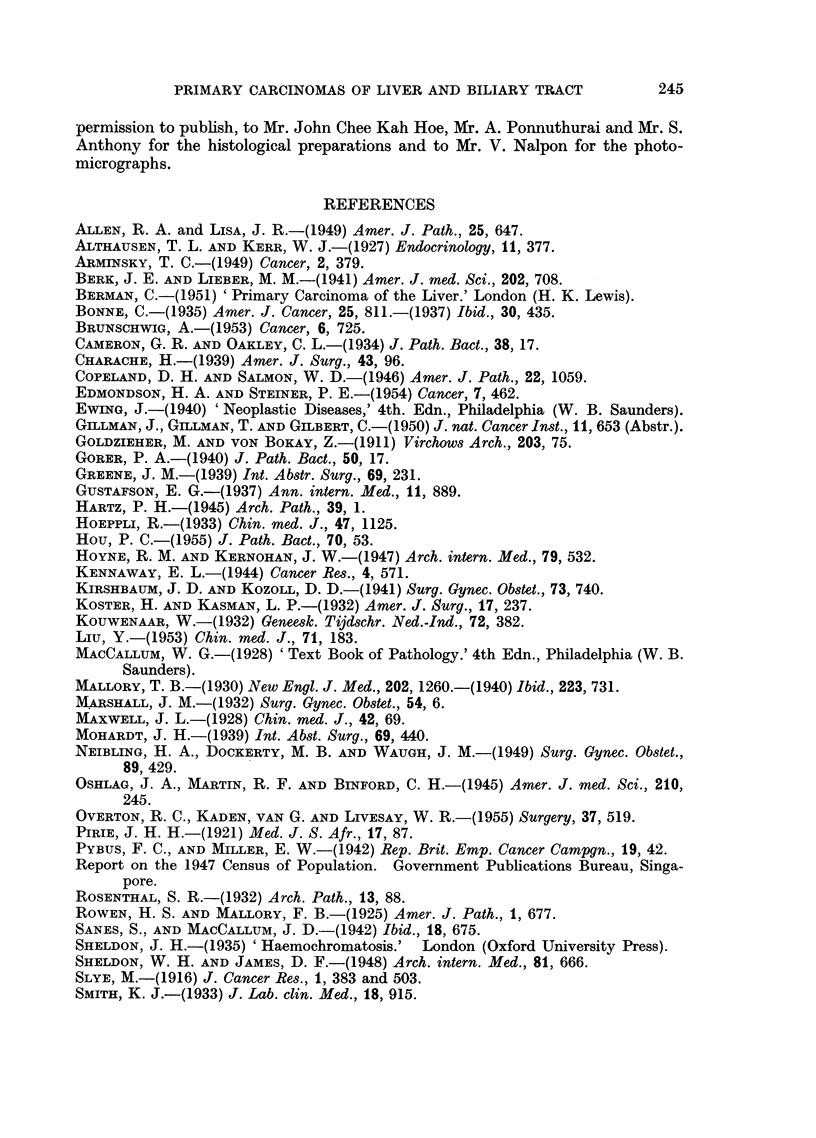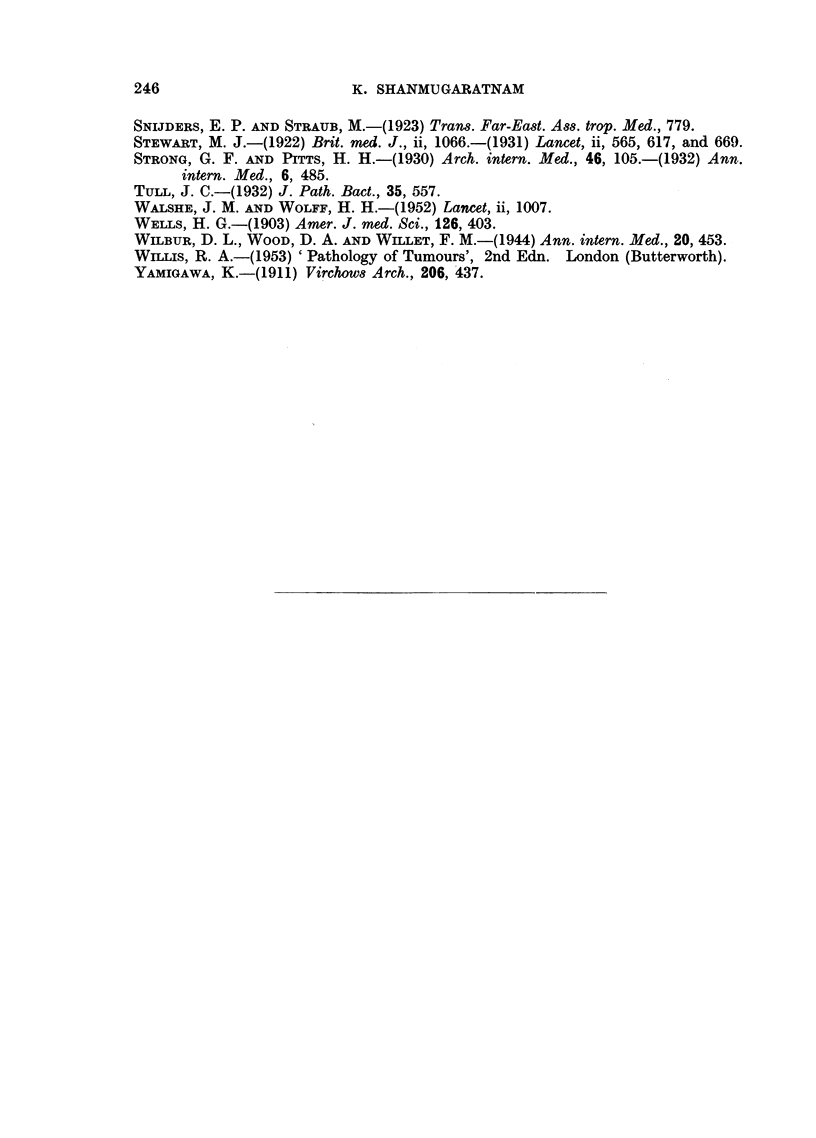# Primary Carcinomas of the Liver and Biliary Tract

**DOI:** 10.1038/bjc.1956.27

**Published:** 1956-06

**Authors:** K. Shanmugaratnam

## Abstract

**Images:**


### PRIMARY CARCINOMAS OF THE LIVER AND BILIARY TRACT

K. SHANMUGARATNAM

*From the Department of Pathology, Singapore*

Received for publication February 20, 1956.

THE classification of carcinomas arising in the liver into liver cell carcinoma and carcinoma of the intra-hepatic bile ducts was put forward by Goldzieher and von Bokay (1911) and supported by Yamigawa (1911). In the literature that has since appeared differences between these tumours in their behaviour, incidence and possible causative factors have not been sufficiently investigated or appreciated for them to be regarded as separate tumours. There has been a tendency to regard them as histological variants of a homogeneous group of tumours and to group them on a regional basis as carcinoma of the liver. Tumours of the liver have attracted special interest on account of their geographical and racial distribution.

This paper is based on 105 cases of carcinoma of the liver and biliary tract found among 9893 post-mortem examinations during a 6½ year period in Singapore, where, as in other parts of South-East Asia and South China, liver cell carcinoma occurs with alarming frequency. In presenting a summary of the pathological evidence gathered from this series, I shall endeavour to illustrate significant differences between carcinomas derived from the liver cells and bile ducts and to strengthen the view that the distribution of the disease is dependent on environmental rather than genetic factors.

The tumours belonged to the following groups :

| | |
|---|---|
| Liver cell carcinoma (hepatocellular carcinoma) . . . . . | 86 cases |
| Carcinoma of the intra-hepatic bile ducts (cholangiocellular carcinoma) | 8 cases |
| Carcinoma of the extra-hepatic bile ducts . . . . . . . | 5 cases |
| Carcinoma of the gall bladder . . . . . . . . . . | 6 cases |

### *Microscopic Features and Diagnostic Criteria*

### *Liver cell carcinoma*

The cytological appearance, the architectural pattern and the stromal reaction are usually distinctive. The tumour cells are usually polygonal and fairly large and have a resemblance to liver cells ; the cytoplasm is abundant and granular and may occasionally contain droplets of bile or globules of fat. Cellular pleomorphism is frequently displayed and giant cells may dominate the picture. A trabecular structure consisting of anastomosing columns of cells reminiscent of the corded architecture of the normal liver is a striking microscopic feature which is nearly always evident if sufficient tissue is examined. The stroma is scanty and typically consists of little more than an endothelial lining investing the tumour cords and forming an " open " vascular network.

Structural variations are common : parts of the tumour may exhibit an alveolar pattern, there may be widespread areas of disorderly or " sarcomatous " structure and there may be a variable fibrous tissue stroma. Although individual characteristics preponderated in some tumours, they did not appear to represent any fundamental difference or to provide a satisfactory basis either to subdivide the tumour into a number of histological types or to grade them according to degree of malignancy.

The exhibition of an alveolar pattern by liver cell carcinoma has been widely accepted (Stewart, 1931 ; Ewing, 1940 ; Berman, 1951 ; Edmondson and Steiner, 1954), but although Tull (1932) is perhaps the only author to deny this the histologic discriptions in literature suggest that carcinomas of the liver have sometimes been classified as cholangiocellular carcinoma because a variable portion had shown an alveolar structure. It is therefore emphasised that an alveolar structure occurs frequently in liver cell carcinoma. It was found in 29 cases in this series : cleft-like spaces were seen in many tumour cords and these could be traced into well developed lumina ; some tumours consisted largely of alveoli lined by single layers of cells.

*Carcinomas of the biliary tract*

*Carcinoma of the intra-hepatic ducts.*—These tumours are usually schirrous adenocarcinomas but may display papillary, mucoid or anaplastic features. The cells are cubical or columnar, resemble biliary epithelium and lack the granularity and occasional eosinophilic character shown by liver cell tumours. Bile formation is not exhibited but necrotic tumours may show some biliary impregnation from without. All the tumours in this series presented an alveolar appearance ; there was evidence of mucus secretion in three cases and in one there was squamous metaplasia. The necropsy findings in the case with squamous metaplasia were those of a large primary hepatic tumour with involvement of the extra-hepatic portion of the right hepatic duct, and numerous liver flukes (*Clonorchis sinensis*) were found in the intra-hepatic ducts. Metastases were found in the lymph nodes at the porta hepatis, the myocardium of the left ventricle and the subpleural tissues of both lungs ; the respiratory passages and mediastinal nodes were normal. Microscopically, the hepatic tumour and all the metastases showed an adeno-carcinomatous structure intimately mixed with areas of squamous-celled structure with " nest " formation and areas of transitional appearance. This microscopic variant of cholangiocellular carcinoma has not been previously described but Mallory (1930), reporting a case of squamous cell carcinoma where an enormous tumour of the liver was associated with tumour involvement of the upper quarter of the common bile duct and retro-peritoneal lumph nodes, made a diagnosis of carcinoma of the extra-hepatic duct because this was the only organ among those involved where a squamous carcinoma was known to occur.

*Carcinoma of the extra-hepatic ducts and gall bladder.*—The majority of these tumours are infiltrating adenocarcinomas with schirrous, mucus-producing and papillary varieties ; anaplastic and squamous varieties are less common. In this series, three of the carcinomas of the extra-hepatic ducts were schirrous adeno-carcinomas with mucoid changes in two of them, one was a diffusely infiltrating signet-ring cell carcinoma and one an anasplastic spheroidal cell carcinoma. Among the gall bladder carcinomas there were three columnar cell adenocarcinomas with mucoid changes, two schirrous adenocarcinomas and one anaplastic carcinoma.

There is no essential histologic difference between carcinomas arising from different parts of the biliary tract. These tumours are not distinguishable microscopically but it has been observed in studies of large series of cases that dense fibrosis is more common in carcinomas of the extra-hepatic ducts and that mucoid, papillary and metaplastic features are more commonly associated with those of the gall bladder.

*Hepatic tumours with intermediate or mixed features*

Cases of hepatocellular and cholangiocellular carcinoma may rarely be found where the typical sharply contrasting microscopic features are not evident and are replaced by intermediate or mixed features. Such tumours have been described by several writers (Wells, 1903 ; Koster and Kasman, 1932 ; Bonne, 1937 ; Gustafson, 1937 ; Mallory, 1940 ; Ewing, 1940 ; Allen and Lisa, 1949 ; Liu, 1953 ; Edmondson and Steiner, 1954) who have variously designated them " mixed tumour ", " duplex type ", " intermediate type ", " carcinoma of dual origin ", " cholangiohepatoma ", " hepatobiliary carcinoma " and " combined liver cell and bile duct carcinoma ". The recorded cases have either been separate neoplastic masses which occasionally fused in collision or individual mases displaying an intimate mixture of both features. The term " cholangiohepatoma " and its synonyms imply a single neoplasm that is the product of a simultaneous malignant change in the liver cells and bile ducts. It is doubtful if such tumours ever occur and the fact that most of the recorded tumours have been in extensively cirrhotic livers (Ewing, 1940) and in males suggests that many of them are histological variants of liver cell carcinoma ; such a conclusion has indeed been reached by some authors (Liu, 1953 ; Edmondson and Steiner, 1954). Should separate hepatocellular and cholangiocellular tumours occur fortuitously in the same liver it would be preferable to name them individually and to avoid the abovementioned terms even if they have fused in places. Single tumours with mixed features would probably reveal their identity if more tissue is examined microscopically, and in the exceptional instance where the diagnosis remains in doubt it would be preferable to classify the tumour as a hepatic carcinoma of undetermined origin.

### *Gross Pathology*

*Liver cell carcinomas* (86 cases)

The majority of tumours appear as multiple nodules or as massive growths measuring from 10–20 cm. in diameter with a few outlying nodules ; less often they appear as poorly defined infiltrations. They are usually soft or pulpy, become crumbling and pultaceous with the onset of massive necrosis and present a variegated appearance due to haemorrhage and biliary impregnation. The hepatic surface commonly presents bosses of tumour tissue which in some instances may even be larger than the rest of the liver and project down to the pelvic brim. Rupture of the tumour with intra-abdominal haemorrhage is a frequent termination (19 cases in this series) ; in exceptional cases this may be the first clinical manifestation of the disease. With few exceptions the liver is appreciably enlarged ; in this series the weights of the liver ranged from 870 g. to 6805 g. with an average weight of 2730 g.

The site of origin : The tumours are usually widely disseminated throughout the liver and it is often difficult to decide on the primary focus of origin. In his monograph on hepatic carcinoma, Berman (1951) recorded a remarkably high tumour incidence in the right lobe and stated that it was highly probable that carcinogenic agents absorbed from the small intestine may be " streamlined "

through the portal vein into the right lobe of the liver. The findings in the present series which are set out below do not support this view :

| | |
|---|---|
| Tumours involving right lobe more extensively | 39 |
| Tumours involving left lobe more extensively | 12 |
| Tumours distributed evenly throughout the organ | 35 |
| Tumours restricted to the right lobe | 15 |
| Tumours restricted to the left lobe | 3 |

The distribution of the tumour in the liver approximates roughly with the relative bulk of the two lobes and does not indicate that the cells in the right lobe are especially prone to develop carcinoma. A comparison of tumours restricted to each lobe shows a relatively greater disparity as the much larger size of the right lobe permits a tumour to be confined to it even after considerable growth.

Changes in the liver : Portal cirrhosis of varying severity was found in 81 (94 per cent) cases in this series ; two of them were cases of hepatic schistosomiasis and one of haemochromotosis. There were no significant abnormalities in the biliary tract.

Growth and metastases : Direct infiltration of the liver was seen in all the cases in this series and in 10 cases there was invasion of the adherent diaphragm. Gross tumour extensions into branches of the portal vein and, less frequently, the hepatic veins were present in 70 (81 per cent) cases and constituted one of the most striking macroscopic features. The main trunk of the portal vein was often distended with tumour tissue and blood clot ; in 6 cases the tumour extended into the inferior vena cava and in three of these it extended further into the cavity of the right atrium. There were frequent and widespread intra-hepatic metastases but blood-borne extra-hepatic metastases were found in only 28 (32 per cent) cases (lungs in 25, stomach in 3, pancreas in 1). Lymph nodal metastases restricted to the abdomen were found in 12 (14 per cent) cases and peritoneal metastases in 2 cases.

The relative infrequency of extra-hepatic metastases in hepatocellular carcinoma is of interest in view of the extreme venous invasion that is often demonstrable and the readiness with which carcinomas of the biliary tract metastasize. In considering this difference, which has been reported previously (Ewing, 1940 ; Hoyne and Kernohan, 1947 ; Edmondson and Steiner, 1954) and is probably due to an inherent character of the tumour cells and their environmental requirements, it may be pertinent to quote the experimental results of Cameron and Oakley (1934) who observed an analogous difference between liver cells and duct cells. In a study of autoplastic transplants of the liver in rats they found that whereas the bile ducts successfully maintained their existence for a long time the liver cells soon began to degenerate and finally dissappeared.

Clinical features : The main symptoms are abdominal pain, abdominal swelling and asthenia and the main physical signs are emaciation, hepatomegaly, ascites, engorged abdominal veins and jaundice. A clinical diagnosis was made in most cases in this series but it was sometimes obscured by the absence of hepatomegaly and when the picture was overshadowed by such complications as abdominal haemorrhage, haematemesis, hepatic coma and intercurrent illnesses. Metastatic symptoms and pyrexia have predominated in some recorded cases. The disease runs an extremely rapid course ; this is due mainly to rapid tumour growth and partly to the " reserve " of the liver which permits a tumour to remain latent until it has grown considerably. Most of the cases in this series (92 per cent) died within 6 months of the onset of symptoms. The following causes of death were established at necropsy :

| | |
|---|---|
| Cachexia with varying degrees of liver failure and venous obstruction . | 50 cases |
| Rupture of tumour with abdominal haemorrhage . . . . . | 19 ,, |
| Rupture of oesophageal varices with haemorrhage . . . . . | 5 ,, |
| Tumour invasion of inferior vena cava and extension into right atrium . | 3 ,, |
| Pulmonary artery occlusion by tumour emboli . . . . . . | 1 ,, |
| Terminal pneumonia . . . . . . . . . . . . | 2 ,, |
| Other diseases . . . . . . . . . . . . | 3 ,, |
| Suicide . . . . . . . . . . . . . . | 3 ,, |

*Carcinomas of the biliary tract* (19 cases)

*Carcinoma of the intra-hepatic ducts.*—These tumours may also assume the form of massive, multinodular or diffusely infiltrating growths but are characteristically firm and homogeneous and do not exhibit the soft texture, extensive necrosis and variegated appearance of hepatocellular carcinoma (Snijders and Straub, 1923 ; MacCallum, 1928 ; Ewing, 1940). They show a greater tendency to involve the hilar portion of the liver and the gross appearances may suggest duct origin : in one of the cases in this series there was localised hydrohepatosis following malignant obstruction of an intra-hepatic duct, in another there was polypoidal tumour extension into the duct lumen and in a third there were warty tumour nodules in the wall of a bile duct cyst associated with tumour nodules in the liver. Five of the tumours involved the right lobe more extensively and three the left.

Changes in the liver : Portal cirrhosis was demonstrated in only one case. The intra-hepatic ducts contained calculi in 3 cases and liver flukes (*C. sinensis*) in one case.

Growth and metastases : The massive venous invasion characteristic of hepatocellular carcinoma is not found but metastases are relatively frequent and widespread. In this series there were blood-borne metastases in five of the eight cases (lungs 5, pancreas 1, kidney 2, heart 2, bones 3, dura mater 2). Spread along the lymphatics and bile ducts may lead to involvement of the extra-hepatic ducts ; this was found in 3 cases.

The clinical features resemble those of hepatocellular carcinoma but it may be observed that the symptoms of abdominal haemorrhage and extreme venous invasion are not features of cholangiocellular carcinoma. They are as rapidly fatal as liver cell tumours ; all the cases in this series died within 6 months of onset of symptoms, death being attributed to cachexia (3 cases), metastases (2 cases), terminal pneumonia (1 case) and other diseases (2 cases).

The macroscopic appearances and behaviour of cholangiocellular carcinoma were found to resemble those of carcinoma of the extra-hepatic biliary tract which are usually diffusely infiltrating schirrous tumours and may exhibit polypoid or mucoid characteristics. The hepatic changes depend on the situation and duration of the tumour and jaundice is frequently the major symptom. Direct invasion of the liver and widespread blood-borne metastases are frequently found.

### *Incidence and Causative Factors*

*Geography and race*

Berman (1951) presented a detailed comparison of the incidence of hepatic carcinoma in various countries and races based on necropsy records. Although variations in the composition of different necropsy series render such a comparison unreliable necropsy figures do provide an approximate guide and uphold the generalisation that carcinoma of the liver is more frequent among the peoples of Asia and Africa than in those of Europe and America.

The composition of the present necropsy series and the distribution of the tumours are shown in Tables I and II. Liver cell carcinoma was present in 0·88 per cent of all necropsies, carcinoma of the intra-hepatic ducts in 0·08 per cent, carcinoma of extra-hepatic ducts in 0·05 per cent and carcinoma of the gall bladder in 0·06 per cent. These percentages are low on account of the selected nature of the necropsies : a large proportion (41 per cent) was on children below 10 years and approximately half the total necropsies were on Coroners' cases. Liver cell carcinoma was the most frequent malignant tumour among necropsies and constituted 20 per cent of all carcinomas ; records of biopsy examinations, however, show that carcinomas of the cervix uteri and nasopharynx are both more frequent in Singapore.

TABLE I.—*Age, Race, and Sex Distribution of this Necropsy Series*

| Age. | Chinese. | | Indians and Pakistanis. | | Malaysians. | | Others. | | Total. | |
|---|---|---|---|---|---|---|---|---|---|---|
| (years.) | M. | F. | M. | F. | M. | F. | M. | F. | M. | F. |
| 0–9 | 2172 | 1657 | 66 | 52 | 32 | 25 | 18 | 5 | 2288 | 1739 |
| 10–19 | 355 | 167 | 27 | 19 | 32 | 7 | 7 | 1 | 421 | 194 |
| 20–29 | 496 | 203 | 106 | 23 | 80 | 12 | 36 | 8 | 718 | 246 |
| 30–39 | 781 | 251 | 184 | 30 | 48 | 9 | 31 | 6 | 1044 | 296 |
| 40–49 | 910 | 228 | 157 | 10 | 34 | 8 | 30 | 8 | 1131 | 254 |
| 50–59 | 733 | 121 | 106 | 8 | 23 | 7 | 34 | 3 | 896 | 139 |
| 60–69 | 308 | 57 | 24 | 7 | 3 | 2 | 9 | 3 | 344 | 69 |
| Over 70 | 67 | 32 | 3 | 1 | 3 | 2 | 5 | 1 | 78 | 36 |
| Total | 5822 | 2716 | 673 | 150 | 255 | 72 | 170 | 35 | 6920 | 2973 |
| | 8538 (86·3%) | | 823 (8·32%) | | 327 (3·31%) | | 205 (2·07%) | | 9893 | |

TABLE II.—*Distribution of Tumours*

| | Male. | Female. | Total. | Chinese. | Indians and Pakistanis. | Others. |
|---|---|---|---|---|---|---|
| Liver cell carcinoma | 81 | 5 | 86 | 82 | 4 | — |
| Carcinoma of the intrahepatic bile ducts | 8 | — | 8 | 7 | 1 | — |
| Carcinoma of the extra-hepatic bile ducts | 5 | — | 5 | 5 | — | — |
| Carcinoma of the gall bladder | 6 | — | 6 | 5 | 1 | — |
| Total carcinomas | 348 | 78 | 426 | 394 | 22 | 10 |
| Total malignant tumours | 416 | 112 | 528 | 478 | 31 | 19 |
| Total necropsies. | 6920 | 2973 | 9893 | 8538 | 823 | 532 |

The frequency of hepatic carcinoma among Oriental and African peoples appears to be largely due to the high frequency of liver cell carcinoma ; it cannot be shown that cholangiocellular carconoma occurs more frequently among these peoples. In the U.S.A. the following ratios of hepatocellular to cholangiocellular carcinomas have been recorded: Smith (1933)—19 : 6 (3·2 : 1); Gustafson (1937)—39 : 21 (1·9 : 1); Wilbur, Wood and Willet (1944)—45 : 4 (11·3 : 1); Hoyne and Kernohan (1947)—20 : 11 (1·8 : 1); Edmondson and Steiner (1954)—75 : 25 (3·0 : 1); Overton, Kaden and Livesay (1955)—49 : 12 (4·1 : 1). In contrast the recorded ratios among the Bantus have been 28 : 3 (9·3 : 1) in the series of Pirie (1921) and 56 : 4 (14·0 : 1) in that of Berman (1951). Similarly, Snijders and Straub (1923) found a ratio of 79 : 4 (19·8 : 1) among the Chinese and Javanese in Sumatra, and in the present series the ratio was 82 : 7 (11·7 : 1) among the Chinese. Tull (1932) recorded a ratio of 99 : 35 (2·8 : 1) in a series from Singapore but this high proportion of cholangiocellular carcinoma is almost certainly due to his non-acceptance of any tendency of liver cell carcinoma to assume an alveolar pattern.

The predisposition of certain races to develop hepatic carcinoma raises the question of whether it is due to genetic factors or to environmental factors or to both. The high incidence of hepatic carcinoma in certain strains of laboratory mice bred from an ancestry with hepatic tumours (Slye, 1916; Gorer, 1940; Pybus and Miller, 1942) has lent support to the theory that the tendency to develop the disease is inheritable. There has, however, been no real evidence of such an inherited tendency in humans in whom the controlling influence of environmental conditions is shown by the relatively low incidence of hepatic carcinoma in the

---

### EXPLANATION OF PLATES

*Liver cell carcinoma*

FIG. 1.—Portion of tumour showing lobular structure closely resembling that seen in portal cirrhosis, but with no interlobular ducts. × 20.
FIG. 2.—Typical arrangement of tumour cells in anastomosing cords. × 85.
FIG. 3.—Thick tumour columns separated by endothelium-lined spaces. × 85.
FIG. 4.—Canalisation of tumour columns. × 85.
FIG. 5.—Deeply stained plugs of bile in several tumour alveoli. × 85.
FIG. 6.—Tumour cells showing some cytological resemblance to normal liver cells. × 340.
FIG. 7.—Cellular pleomorphism in anaplastic tumour; several multinucleated tumour cells. × 340.

*Carcinoma of the intra-hepatic bile ducts*

FIG. 8.—Tumour showing adenocarcinomatous structure with some adjoining normal liver tissue. × 85.
FIG. 9.—Tumour alveoli separated by dense fibrous stroma. × 85.
FIG. 10.—Cytological appearance of tumour shown in Fig. 9. × 340.
FIG. 11.—Tumour showing metaplastic squamous cell features in addition to adenocarcinomatous structure. × 85.
FIG. 12.—Another portion of tumour shown in Fig. 11 exhibiting a predominantly squamous structure; a zone of surviving liver tissue is seen in the middle. × 85.

*Liver cell carcinoma*

FIG. 13.—" Massive " variety of liver cell carcinoma.
FIG. 14.—" Multinodular " variety of liver cell carcinoma; extensive filling of branches of portal vein with tumour tissue.
FIG. 15.—" Diffusely infiltrating " variety of liver cell carcinoma.

*Carcinoma of the intra-hepatic bile ducts*

FIG. 16.—Warty tumour nodules in wall of bile duct cyst associated with conglomerate tumour nodules in one half of liver; a small white probe has been placed in duct leading from cyst.
FIG. 17.—Tumour of right lobe of liver extending into lumen of right hepatic duct.

1

2

3

4

5

6

Shanmugaratnam.

7

8

9

10

11

12

13

14

15

16

17

American Negro (Kennaway, 1944) and the distribution of the disease in Africa (Gillman, Gillman and Gilbert, 1950).

Chinese patients have figured prominently in series of hepatic carcinoma in Western countries but most of them have been China-born immigrants who have been in their new environment for relatively short periods. The high incidence among Chinese in this series was restricted to the immigrants and those born in Singapore showed no undue susceptibility to the disease (Table III). Of the 73 cases of liver cell carcinoma where information regarding the place of birth was available only one was born in Singapore, four were born in neighbouring countries and the remaining 68 cases (93 per cent) occurred among those born in China, all of whom were from the southern provinces of Kwangtung and Fukien and the island of Hainan off the South China coast. An overwhelming majority of the Chinese immigrants in Singapore are also from these parts of South China. All four Indians with liver cell carcinoma were born in India.

A relatively greater number of Singapore-born Chinese belong to the lower age groups and, as 95 per cent of liver cell carcinomas occurred above the age of 30 years, the relatively high incidence among China-born Chinese in Table III is also due to selection. The age distribution of the census figures is not available but that of 1000 consecutive Chinese patients is provided in Table IV for compari-

TABLE III.—*An Analysis of the Place of Birth of Cases of Carcinoma in Chinese where such information was available, of* 1000 *consecutive Chinese Patients and the Chinese Population of Singapore*

| | Total. | Number born in Singapore. | Number born in China. | Number born elsewhere. |
|---|---|---|---|---|
| Chinese population in Singapore (1947 census) | 73,013 | 421,406 (57·72%) | 281,032 (38·49%) | 27,695 (3·79%) |
| Consecutive admissions to medical wards | 1,000 | 541 | 438 | 21 |
| Liver cell carcinoma | 73 | 1 | 68* | 4 |
| Carcinoma of the intra-hepatic bile ducts | 7 | — | 7 | — |
| Carcinoma of the extra-hepatic bile ducts | 5 | — | 5 | — |
| Carcinoma of the gall bladder | 3 | — | 3 | — |

* The period since arrival in Singapore was stated to be less than 10 years in 8 cases, 10–19 years in 15 cases, 20–29 years in 14 cases, 30–39 years in 14 cases, 40–49 years in 4 cases and was unknown in 13 cases.

TABLE IV.—*The Age and Place of Birth of the* 1000 *Consecutive Chinese Patients Referred to in Table III*

| Age. | Number of patients born in Singapore. | Number of patients born in China. | Average period since arrival in Singapore. | Number of patients born elsewhere. |
|---|---|---|---|---|
| 0–9 | 64 (98·5%) | 0 | — | 1 |
| 10–19 | 179 (95·2%) | 6 | 6·3 yrs. | 3 |
| 20–29 | 129 (73·7%) | 44 | 11·4 yrs. | 2 |
| 30–39 | 81 (50·0%) | 71 | 12·8 yrs. | 10 |
| 40–49 | 45 (27·3%) | 116 | 14·3 yrs. | 4 |
| 50–59 | 26 (17·7%) | 121 | 22·4 yrs. | — |
| 60–69 | 15 (18·8%) | 64 | 24·4 yrs. | 1 |
| 70 and over | 2 (11·1%) | 16 | 28·8 yrs. | — |
| Total | 541 (54·1%) | 438 | — | 21 |

son. The ratio of China-born to Singapore-born Chinese was 2·3 : 1 among patients over 30 years but there were 68 liver cell carcinomas and 15 carcinomas of the biliary tract among China-born Chinese and only one liver cell carcinoma occurred in a Singapore-born Chinese. These figures indicate that the difference between the two groups, although exaggerated in Table III, is nevertheless significant. As there is hardly any genetic difference between the immigrant Chinese and the indigenous Chinese of Singapore of whom a great majority have been in Singapore for less than 3 to 4 generations, these findings indicate that the occurrence of the tumour was not due to any inherited susceptibility but to some difference in the environment or mode of life to which these groups of people have been subjected.

*Age*

The cases of liver cell carcinoma ranged from 19 years to 74 years with a maximum frequency in the fifth decade (43 per cent of cases) followed by the sixth decade (30 per cent of cases) and the fourth decade (14 per cent of cases). A liver cell carcinoma found in an infant aged 13 months was considered to be probably a hepatoblastoma and excluded from this series.

The age distribution of carcinoma of the intra-hepatic ducts was between 40 and 70 years, that of carcinoma of the extra-hepatic ducts between 37 and 60 years and that of carcinoma of the gall bladder between 45 and 68 years. Ewing (1940) observed that cholangiocellular carcinoma tends to occur at later ages than liver cell carcinoma.

*Sex*

An overwhelming majority of liver cell carcinomas occurs in males. In this series males constituted 93 per cent of all cases of liver cell carcinoma but only 70 per cent of all necropsies. The tumour was found in 1·2 per cent of necropsies on males and 0·2 per cent of necropsies on females. It is evident from these and all other published figures that the marked male predominance is due to a true sex predilection in addition to the preponderance of males in most necropsy series. Furthermore, it has been observed that male animals are more prone to develop experimentally produced liver cell carcinoma.

On the other hand, accounts in the literature indicate that females are more frequently affected by carcinomas of the biliary tract. In this series, however, all the carcinomas of the biliary tract occurred in males, but in view of the small numbers of these tumours and the fact that more than 80 per cent of necropsies above the age of 30 years were on males, no conclusion is drawn regarding sex predisposition. The decided frequency of carcinoma of the gall bladder among females is well known (Mohardt, 1939 ; Arminsky, 1949). Carcinoma of the extra-hepatic ducts has been recorded more frequently in males but published figures show that the sexes are almost equally susceptible (Kirshbaum and Kozoll, 1941 ; Neibling, Dockerty and Waugh, 1949 ; Willis, 1953). An analysis of recorded cases indicates that carcinoma of the intra-hepatic ducts occurs more frequently in females (Gustafson, 1937 ; Hoyne and Kernohan, 1947 ; Charache, 1939 ; Ewing, 1940 ; Edmondson and Steiner, 1954).

*Portal cirrhosis*

Portal cirrhosis is the most frequent precancerous lesion associated with liver cell carcinoma ; it is not commonly associated with carcinomas of the biliary tract. In this series it was present in 81 (94 per cent) cases of liver cell carcinoma, one case of carcinoma of the intra-hepatic ducts, one of carcinoma of the extra-hepatic ducts and none of carcinoma of the gall bladder. The striking difference between the incidence of cirrhosis in hepatocellular and cholangiocellular carcinomas has been acknowledged by many writers (Goldzieher and Bokay, 1911; Smith, 1933; Stewart, 1931; Snijders and Straub, 1923; Liu, 1953; Willis, 1953; Gustafson, 1937; Greene, 1939; Edmondson and Steiner, 1954).

The frequency of carcinomatous change in cirrhotic livers was found to be 3·4 per cent by Stewart (1931) in a series from England and 9·3 per cent by Rosenthal (1932) in a series from America; in an analysis of 1989 cases of cirrhosis reported by different authors in Europe and America, Berk and Lieber (1941) found carcinomatous change in 4·5 per cent. Liu (1953) observed malignant transformation in 12·6 per cent of cases of cirrhosis in a series from China and Kouwenaar (1932) estimated that it occurred in 20 to 25 per cent of cirrhotic livers among the Javanese.

From the findings in this series, shown in Table V, several observations may be made. Firstly, cirrhosis is shown to be relatively prevalent, being demonstrable

TABLE V.—*The Incidence of Cirrhosis and the Frequency of Associated Liver Cell Carcinoma in this Necropsy Series*

| | Frequency of cirrhosis in total necropsies. | Frequency of cirrhosis in necropsies over 30 years. | Frequency of liver cell carcinoma in cases of cirrhosis. |
|---|---|---|---|
| *Chinese* | | | |
| Males | 257 (4·4%) | 240 (8·6%) | 73 (28·4%) |
| Females | 44 (1·6%) | 38 (5·5%) | 5 (11·4%) |
| Total | 301 (3·5%) | 278 (8·0%) | 78 (25·9%) |
| *Indians and Pakistanis* | | | |
| Males | 31 (4·6%) | 26 (5·5%) | 3 (9·7%) |
| Females | 3 (2·0%) | 3 (5·4%) | — |
| Total | 34 (4·1%) | 29 (5·5%) | 3 (8·8%) |
| *Malaysians* | | | |
| Males | 9 (3·5%) | 9 (8·1%) | — |
| Females | 1 (1·4%) | 1 (3·6%) | — |
| Total | 10 (3·1%) | 10 (7·2%) | — |
| *Others* | | | |
| Males | 7 (4·1%) | 7 (6·4%) | — |
| Females | 1 (2·9%) | — | — |
| Total | 8 (3·9%) | 7 (5·4%) | — |
| *Total* | | | |
| Males | 304 (4·4%) | 282 (8·1%) | 76 (25·0%) |
| Females | 49 (1·7%) | 42 (5·3%) | 5 (10·2%) |
| Total | 353 (3·6%) | 324 (7·6%) | 81 (23·0%) |

in 3·6 per cent of all necropsies and 7·6 per cent of necropsies over the age of 30 years. Secondly, there was an unusually high rate of malignant transformation in cirrhotic livers especially among the Chinese; 23 per cent of all cases of cirrhosis showed liver cell carcinoma. Thirdly, malignant transformation in cases of cirrhosis was decidely more frequent in males (25 per cent) than females (10·2 per cent). Edmondson and Steiner (1954) have also observed that women with cirrhosis are less likely to develop liver cell carcinoma.

*Infective hepatitis*

Sheldon and James (1948) and Walshe and Wolff (1952) have recorded cases of liver cell carcinoma following hepatitis. Hepatitis is a fairly common disease in Singapore but there is no accurate information regarding its incidence.

*Haemochromatosis*

The association of liver cell carcinoma with haemochromatosis is well known, and although some authors have held the view that this is due to the coexistent cirrhosis (Stewart, 1922; Rosenthal, 1932; Sheldon, 1935), the consensus of opinion is that it is more frequent than in uncomplicated cirrhosis (Rowen and Mallory, 1925; Althausen and Kerr, 1927; Gustafson, 1937; Berk and Lieber, 1941). The only case of haemochromatosis in this series was associated with liver cell carcinoma.

Carcinoma of the intra-hepatic ducts, on the other hand, is not associated with haemochromatosis; the only case on record is by Oshlag, Martin and Binford (1945) but their histologic description that the tumour consisted of cords and acini of large polyhedral cells arranged in a lobular pattern somewhat like that of pre-existing liver is typical of liver cell carcinoma.

*Dietary factors*

The distribution of hepatic carcinoma among peoples in whom malnutrition is common, and the observation that hepatic disease may follow dietary deficiencies and the oral administration of toxic substances have led to the supposition that the main causal factor in the production of liver cell carcinoma is dietary. This view is strengthened by Copeland and Salmon's (1946) observation that deficient diets may predispose to hepatic carcinoma in experimental animals. Such a relationship may well play a part in man but has not been proved.

*Cholelithiasis*

There is a close association of gall stones with carcinoma of the gall bladder (Mohardt, 1939; Arminsky, 1949) and, to a lesser extent,with carcinoma of the extra-hepatic ducts (Marshall, 1932; Ewing, 1940; Kirshbaum and Kozoll, 1941; Neibling, Dockerty and Waugh, 1949); in both instances the occurrence of calculi is much greater than in any control group of cases and is probably related to the development of carcinoma.

Cholelithiasis is exceptionally rare in liver cell carcinoma but is more frequently associated with carcinoma of the intra-heaptic ducts. Sanes and MacCallum (1942) reported two cases of cholangiocellular carcinoma associated with hepatolithiasis and put forward the view that a calculous cholangitis was a possible causative factor; Brunschwig's (1953) case of a hepatic carcinoma in a woman who had an operation for calculi was also stated to be a cholangiocellular carcinoma. In this series gall stones were present in the gall bladder in only 2 (2·3 per cent) cases of liver cell carcinoma but were present in 3 of the 8 cases of carcinoma of the intra-hepatic ducts, in two of which they were located within the liver in association with the primary growth.

*Parasites*

A causal relationship between hepatic carcinoma and parasitic infestations of the liver has been supported in view of the high frequency of schistosomiasis in

South Africa (Pirie, 1921) and clonorchiasis in South China (Maxwell, 1928; Strong and Pitts, 1930 and 1932; Liu, 1953). It is not unreasonable to suppose that parasites may predispose to tumour growth but the existing evidence in favour of such a relationship is insufficient and consists only of a certain similarity in the geographical distribution of both diseases and their occasional coexistence in the same case. In Java and Sumatra, where schistosomiasis is not indigenous, helminthic infestations have played no part in the incidence of hepatic carcinoma (Bonne, 1935; Hartz, 1945). Hoeppli (1933) studied 66 cases of clonorchiasis and found only one case with portal cirrhosis and none with either carcinoma or any change that could be regarded as a precancerous stage. In a study of 500 cases of clonorchiasis, Hou (1955) found no direct relationship between parasitic infestation and portal cirrhosis but observed that a malignant change in the bile ducts was one of the most serious consequences of the disease.

Clonorchiasis and schistosomiasis are unknown among the indigenous people of Singapore who have not been shown to be prone to develop hepatic carcinoma. Among the immigrant Chinese in this series there were 8 cases of *S. japonicum* infestation of the liver in two of which there was associated liver cell carcinoma, and 67 cases of *C. sinensis* infestation among which there was one case of cholangiocellular carcinoma and none of liver cell carcinoma.

### Discussion

An analysis of the findings in this series together with data culled from literature shows that there are several significant differences between liver cell carcinoma and carcinoma of the intra-hepatic bile ducts besides their microscopic features. Liver cell carcinoma occurs predominantly in males and especially among certain Oriental and African peoples. Portal cirrhosis is found in a majority of cases and appears to be the most significant amoug the various predisposing factors that have been suggested; further investigation is necessary before the roles of dietary deficiencies, infective hepatitis and parasitic infestations may be assessed. The growth of the tumour is characteristic by extensive venous invasion but extra-hepatic metastases are surprisingly infrequent. On the other hand, carcinoma of the intra-hepatic bile ducts occurs more frequently among females and does not show the racial predilection or the association with portal cirrhosis observed in liver cell carcinoma. It appears possible that the presence of parasites or calculi in the intra-hepatic ducts may be predisposing factors. Furthermore, extra-hepatic metastases are frequent and widespread although gross tumour invasion is not featured.

In all the aforementioned points carcinoma of the intra-hepatic ducts resembles carcinomas of the extra-hepatic biliary tract and is best classified with them. These groups of tumours have similar microscopic appearances and their separation, which is only based on their anatomical location in the biliary tract, is often arbitrary or impossible when there is widespread tumour involvement due to invasive spread or a wide field of origin.

It is accepted, however, that although hepatocellular and cholangiocellular carcinomas ordinarily present sharply contrasting microscopic features, some tumours may display features of a somewhat intermediate or mixed character. Such appearances are mostly due to the capacity of liver cell carcinoma to exhibit variations in microscopic structure, in particular to assume an alveolar or tubular architecture similar to that of cholangiocellular tumours. The argument that such appearances invalidate the histogenetic distinction between these tumours becomes weak when one considers the microscopic variations which every malignant tumour may exhibit and the structural similarity which is sometimes observed in tumours of widely different origin. Carcinoma of the liver poses an additional diagnostic problem occasioned by the existence of two different tumours in the same organ. In supporting the view that hepatocellular and cholangiocellular carcinomas are structural variants of a homogeneous group of tumours, Berman (1951) argued that liver cells and bile ducts have a common embryological derivation, that the adult cells of one type can change to the other and that it is not unexpected that a similar reaction may take place in carcinoma of the liver. However, embryologists have not agreed on the details of hepatic development and the consensus of opinion is that liver cells and bile ducts have a common embryological derivation but are formed from groups of cells that have separated early in foetal life. Furthermore, it is the adult character of the tissues of origin rather than their embryological derivation which determines the behaviour of tumours and on which classification is satisfactorily based. The transformation of one type of cell to the other is a debatable point ; there is no unequivocal evidence of such a change and most writers have agreed that it does not play any significant role in the regeneration of liver tissue. The view that these cells grow independently of each other is supported by experimental findings (Cameron and Oakley, 1934).

The origin of hepatocellular and cholangiocellular carcinomas from the liver cells and bile ducts respectively is abundantly shown by their microscopic appearances, their association with hyperplastic states of their parent tissues and the occasionally demonstrable transition between the tumour cells and their normal precursors. The findings in this series endorsed the importance of intra-hepatic dissemination in the development of multi-nodular tumours but indicated that a genuine multi-focal origin also occurs in many cases. The greater frequency of carcinoma in the right lobe was found to be proportionate to its bulk.

One of the main findings in the present study was the disparity between the immigrant Chinese from South China and the indigenous Chinese of Singapore in their susceptibility to develop liver cell carcinoma. The high tumour incidence in the former group was not observed among the Singapore-born Chinese and, as there is no genetic difference between them, an investigation of changes in their environment or mode of life promises to be fruitful.

### SUMMARY

A summary of the pathological findings in 105 cases of carcinoma of the liver and biliary tract is presented with a review of the histogenesis, causation and distribution of these tumours. It was considered that liver cell carcinoma and carcinoma of the intra-hepatic bile ducts are heterogeneous tumours and that the latter are closely similar to carcinomas of the extra-hepatic biliary tract. It is shown that the high incidence of liver cell carcinoma among the Chinese of Singapore is restricted to the immigrants from South China.

My thanks are due to Dr. W. J. Vickers, Dr. R. H. Bland, Dr. C. Subrahmanyam and Dr. L. S. da Silva, Singapore, for the opportunity to make this study and for permission to publish, to Mr. John Chee Kah Hoe, Mr. A. Ponnuthurai and Mr. S. Anthony for the histological preparations and to Mr. V. Nalpon for the photomicrographs.

### REFERENCES

ALLEN, R. A. and LISA, J. R.—(1949) *Amer. J. Path.*, **25**, 647.
ALTHAUSEN, T. L. AND KERR, W. J.—(1927) *Endocrinology*, **11**, 377.
ARMINSKY, T. C.—(1949) *Cancer*, **2**, 379.
BERK, J. E. AND LIEBER, M. M.—(1941) *Amer. J. med. Sci.*, **202**, 708.
BERMAN, C.—(1951) ' Primary Carcinoma of the Liver.' London (H. K. Lewis).
BONNE, C.—(1935) *Amer. J. Cancer*, **25**, 811.—(1937) *Ibid.*, **30**, 435.
BRUNSCHWIG, A.—(1953) *Cancer*, **6**, 725.
CAMERON, G. R. AND OAKLEY, C. L.—(1934) *J. Path. Bact.*, **38**, 17.
CHARACHE, H.—(1939) *Amer. J. Surg.*, **43**, 96.
COPELAND, D. H. AND SALMON, W. D.—(1946) *Amer. J. Path.*, **22**, 1059.
EDMONDSON, H. A. AND STEINER, P. E.—(1954) *Cancer*, **7**, 462.
EWING, J.—(1940) ' Neoplastic Diseases,' 4th. Edn., Philadelphia (W. B. Saunders).
GILLMAN, J., GILLMAN, T. AND GILBERT, C.—(1950) *J. nat. Cancer Inst.*, **11**, 653 (Abstr.).
GOLDZIEHER, M. AND VON BOKAY, Z.—(1911) *Virchows Arch.*, **203**, 75.
GORER, P. A.—(1940) *J. Path. Bact.*, **50**, 17.
GREENE, J. M.—(1939) *Int. Abstr. Surg.*, **69**, 231.
GUSTAFSON, E. G.—(1937) *Ann. intern. Med.*, **11**, 889.
HARTZ, P. H.—(1945) *Arch. Path.*, **39**, 1.
HOEPPLI, R.—(1933) *Chin. med. J.*, **47**, 1125.
HOU, P. C.—(1955) *J. Path. Bact.*, **70**, 53.
HOYNE, R. M. AND KERNOHAN, J. W.—(1947) *Arch. intern. Med.*, **79**, 532.
KENNAWAY, E. L.—(1944) *Cancer Res.*, **4**, 571.
KIRSHBAUM, J. D. AND KOZOLL, D. D.—(1941) *Surg. Gynec. Obstet.*, **73**, 740.
KOSTER, H. AND KASMAN, L. P.—(1932) *Amer. J. Surg.*, **17**, 237.
KOUWENAAR, W.—(1932) *Geneesk. Tijdschr. Ned.-Ind.*, **72**, 382.
LIU, Y.—(1953) *Chin. med. J.*, **71**, 183.
MACCALLUM, W. G.—(1928) ' Text Book of Pathology.' 4th Edn., Philadelphia (W. B. Saunders).
MALLORY, T. B.—(1930) *New Engl. J. Med.*, **202**, 1260.—(1940) *Ibid.*, **223**, 731.
MARSHALL, J. M.—(1932) *Surg. Gynec. Obstet.*, **54**, 6.
MAXWELL, J. L.—(1928) *Chin. med. J.*, **42**, 69.
MOHARDT, J. H.—(1939) *Int. Abst. Surg.*, **69**, 440.
NEIBLING, H. A., DOCKERTY, M. B. AND WAUGH, J. M.—(1949) *Surg. Gynec. Obstet.*, **89**, 429.
OSHLAG, J. A., MARTIN, R. F. AND BINFORD, C. H.—(1945) *Amer. J. med. Sci.*, **210**, 245.
OVERTON, R. C., KADEN, VAN G. AND LIVESAY, W. R.—(1955) *Surgery*, **37**, 519.
PIRIE, J. H. H.—(1921) *Med. J. S. Afr.*, **17**, 87.
PYBUS, F. C., AND MILLER, E. W.—(1942) *Rep. Brit. Emp. Cancer Campgn.*, **19**, 42.
Report on the 1947 Census of Population. Government Publications Bureau, Singapore.
ROSENTHAL, S. R.—(1932) *Arch. Path.*, **13**, 88.
ROWEN, H. S. AND MALLORY, F. B.—(1925) *Amer. J. Path.*, **1**, 677.
SANES, S., AND MACCALLUM, J. D.—(1942) *Ibid.*, **18**, 675.
SHELDON, J. H.—(1935) ' Haemochromatosis.' London (Oxford University Press).
SHELDON, W. H. AND JAMES, D. F.—(1948) *Arch. intern. Med.*, **81**, 666.
SLYE, M.—(1916) *J. Cancer Res.*, **1**, 383 and 503.
SMITH, K. J.—(1933) *J. Lab. clin. Med.*, **18**, 915.

SNIJDERS, E. P. AND STRAUB, M.—(1923) *Trans. Far-East. Ass. trop. Med.*, 779.
STEWART, M. J.—(1922) *Brit. med. J.*, ii, 1066.—(1931) *Lancet*, ii, 565, 617, and 669.
STRONG, G. F. AND PITTS, H. H.—(1930) *Arch. intern. Med.*, **46**, 105.—(1932) *Ann. intern. Med.*, **6**, 485.
TULL, J. C.—(1932) *J. Path. Bact.*, **35**, 557.
WALSHE, J. M. AND WOLFF, H. H.—(1952) *Lancet*, ii, 1007.
WELLS, H. G.—(1903) *Amer. J. med. Sci.*, **126**, 403.
WILBUR, D. L., WOOD, D. A. AND WILLET, F. M.—(1944) *Ann. intern. Med.*, **20**, 453.
WILLIS, R. A.—(1953) 'Pathology of Tumours', 2nd Edn. London (Butterworth).
YAMIGAWA, K.—(1911) *Virchows Arch.*, **206**, 437.